# Effects of Cold Compress on Pain and Vascular Complications in Patients With Percutaneous Coronary Intervention: A Systematic Review and Meta-Analysis

**DOI:** 10.1097/jnr.0000000000000756

**Published:** 2026-08-13

**Authors:** Yi-Da LI, Ying-Chin LIAO

**Affiliations:** 1 Department of Medical, Dalin Tzu Chi Hospital, Buddhist Tzu Chi Medical Foundation, Chiayi, Taiwan; 2 School of Medicine, Tzu Chi University; 3 Department of Nursing, Dalin Tzu Chi Hospital, Buddhist Tzu Chi Medical Foundation, Chiayi, Taiwan; 4 Department of Nursing, National Cheng Kung University, Tainan, Taiwan

**Keywords:** percutaneous coronary intervention, cold compress, pain, vascular complications

## Abstract

**Background:**

Transfemoral percutaneous coronary intervention (PCI), a widely adopted treatment for coronary artery disease, is frequently accompanied by vascular complications such as pain, bleeding, ecchymosis, and hematoma. Although cold compress application has been suggested to mitigate these complications as a simple, nonpharmacological intervention, the evidence regarding its effectiveness is inconsistent.

**Purpose:**

The goal of this meta-analysis is to evaluate the effectiveness of cold compresses in alleviating pain and preventing vascular complications in patients undergoing transfemoral PCI.

**Methods:**

Four significant databases (Embase, Medline, Cochrane, CINHAL database), gray literature, and the Clinical Trials Registry were searched using the following keywords: P: “transfemoral percutaneous coronary interventions” OR “coronary interventions”; I: “cold pack” OR “cold compress” OR “ice-bag”; and O: “vascular complications” OR “adverse reactions” OR “pain.” Two independent authors screened the studies based on predefined inclusion criteria, and any discrepancies were resolved through discussion. The quality of the included studies was assessed using the Joanna Briggs Institute (JBI) critical appraisal checklist. Data were synthesized using statistical methods to calculate the standardized mean difference (SMD) for pain levels and risk ratios (RR) for outcomes such as hematoma, ecchymosis, and bleeding. This research was registered in the PROSPERO database (CRD420251018936).

**Results:**

Ten randomized controlled trials (RCTs) involving 904 participants were included in the analysis. The results of the pooled analyses showed cold compresses significantly reduced puncture-site pain (SMD=−1.56, 95% CI [−2.86, −0.25]), bleeding (RR=0.30, 95% CI [0.18, 0.52]), and hematoma (RR=0.30, 95% CI [0.10, 0.91]) and demonstrated a detectable but nonsignificant reduction effect on ecchymosis incidence (RR=0.38, 95% CI [0.10, 1.36]). The results of the subgroup analyses revealed consistent protective effects for bleeding and hematoma, although pain and ecchymosis outcomes exhibited moderate-to-high heterogeneity based on specific intervention modality. The methodological quality of the included studies was assessed to be moderate to high (JBI scores: 9–12).

**Conclusions/Implications for Practice:**

The findings support cold compresses as effective in alleviating puncture-site pain and reducing bleeding and hematoma following transfemoral PCI, with a possible beneficial effect on ecchymosis. Despite protocol variability and the absence of blinding across trials, the overall evidence supports the clinical application of cold compresses as a safe, low-cost, and nonpharmacological adjunct to standard post-PCI care. To optimize outcomes, standardized intervention protocols, including timing, temperature, weight, and duration, should be adopted in future trials. Based on the current evidence, the use of a cold pack (100–250 g, maintained at 15°C–20°C, applied for 15–20 min after manual compression) is recommended to enhance patient comfort and recovery in routine nursing practice.

## Introduction

Coronary artery disease (CAD) remains the leading cause of morbidity and mortality worldwide. With advances in medical technology, percutaneous coronary intervention (PCI) has become a standard treatment for CAD as a less-invasive alternative to coronary artery bypass grafting (CABG; Abubakar et al., 2023).

PCI is a minimally invasive procedure used to restore blood flow in narrowed or blocked coronary arteries. It involves the insertion of a catheter into the artery, which is then guided to the coronary arteries, allowing for balloon angioplasty and stent placement to improve vascular patency. PCI has significantly reduced the need for open-heart surgery and improved outcomes and quality of life in patients with acute coronary syndromes and stable ischemic heart disease (Abubakar et al., 2023; Farooqi et al., 2022; Yujeong, 2022). However, this procedure is not without risks, as vascular access complications such as hematoma, ecchymosis, and bleeding are common concerns, particularly in cases of transfemoral PCI (Wennberg et al., 2025). Transfemoral PCI is a procedure widely performed for the management of CAD. Despite its effectiveness, patients undergoing transfemoral PCI are at risk of vascular complications, including hematoma, ecchymosis, and bleeding, primarily due to arterial puncture and postprocedural immobilization. Pain and discomfort following the procedure can further impact patient recovery and overall satisfaction with care (Su et al., 2019; Wennberg et al., 2025). Although both transfemoral and transradial PCI are widely practiced, the analysis in this study was restricted to studies on transfemoral access only. Compared with transradial PCI, transfemoral PCI carries higher risks of bleeding, hematoma, and ecchymosis due to the larger vessel caliber and need for prolonged hemostasis and immobilization (Yamamoto et al., 2020).

Applying cold compresses is one of several strategies that have been investigated with regard to mitigating the complications of PCI (Halm et al., 2022; Mall, 2019). Cold therapy has been recognized for its potential to reduce pain, inflammation, and vascular complications across different clinical settings. Its physiological effects, including vasoconstriction, decreased capillary permeability, and reduced metabolic activity, should theoretically help minimize hematoma formation and alleviate discomfort (Asy’ary et al., 2024; Garcia et al., 2021; Halm et al., 2022). Although the application of cold compresses after transfemoral PCI has been investigated in several studies, the findings have been inconsistent, with some studies reporting significant reductions in pain and vascular complications and others reporting no substantial benefits. A recent systematic review by Sugiharto et al. (2025), which focused mainly on hematoma and pain outcomes in patients undergoing femoral PCI, provided a valuable descriptive synthesis on this topic. However, a meta-analysis was not performed as part of their work due to the heterogeneity in outcome reporting and to outcomes such as ecchymosis and bleeding not being quantitatively synthesized. Furthermore, although variations in intervention modalities (e.g., ice bag with sandbag, early ambulation combined with ice bag) were acknowledged, the impact of this variation on heterogeneity was not formally explored. These discrepancies highlight the need for a comprehensive synthesis of the current evidence. Therefore, the aim of this systematic review and meta-analysis was to evaluate the effectiveness of cold compresses in improving clinical outcomes following transfemoral PCI to enhance patient recovery and safety following transfemoral PCI.

### Study Purpose

This meta-analysis was performed to evaluate the effectiveness of cold compresses in alleviating pain and preventing vascular complications in patients undergoing transfemoral PCI.

## Methods

This study followed the Preferred Reporting Items for Systematic Review and Meta-Analysis (PRISMA) flowchart guidelines and is registered on PROSPERO (Number CRD420251018936). After two researchers reviewed each step independently, cross-checking was performed to ensure study quality and the accuracy of the conclusions made. To address concerns of potential patient overlap among the included studies, the study identifiers, including recruitment year, setting, and sample size, were examined carefully. In the event multiple included studies were conducted in similar hospitals, they were checked to confirm they reported distinct outcomes and enrolled participants during different time frames. In cases of possible overlap, sensitivity analyses were performed by excluding one of the studies to verify finding consistency. All of the abovementioned helped ensure the robustness of the results.

### Search Strategy

The full-text databases of the Cochrane Library, MEDLINE, CINAHL, and Embase were searched on March 28, 2025, for randomized controlled studies (RCTs). The search terms used were determined following discussions with a medical university librarian. The search strategy combined free-text terms and controlled vocabularies, including synonyms and MeSH terms, to comprehensively capture relevant studies. The final Boolean search string was (“percutaneous coronary interventions” OR “transfemoral percutaneous coronary interventions” OR “cardiac catheterization” OR “coronary intervention”) AND (“cold compress” OR “cold pack” OR “ice-bag” OR “cold sand pack” OR “cold therapy”) AND (“vascular complications” OR “adverse reactions” OR “hemostasis” OR “bleeding” OR “ecchymosis” OR “hematoma” OR “pain”). Detailed search strategies for each database are provided in Supplementary File 1, Supplemental Digital Content 1, http://links.lww.com/JNR/A13. Two independent reviewers with ICU experience screened the literature for eligible randomized experimental studies, and any disagreements were resolved by a third reviewer, who was an ICU specialist.

### Inclusion Criteria

Population (P): Adults at least 18 years old diagnosed with CAD requiring a transfemoral percutaneous coronary intervention.

Intervention (I): Cold compress, with water used as the therapeutic agent, classified as cool for temperatures of 18.3 °C–26.7 °C and cold for temperatures of 12.8 °C–18.3 °C (Halm et al., 2022). Cold compresses may be applied either before or after arterial sheath removal.

Comparison (C): The control group comprised patients who received usual care (sandbag).

Outcomes (O): Vascular complications medically defined as hematoma, ecchymosis, or bleeding (assessment made by the researcher). Puncture-site pain was determined by using assessment tools (i.e., Numeric Rating Scale and Visual Analogue Scale).

### Exclusion Criteria

Studies with patients reporting vascular complications at baseline, that were nonrandomized, or that had original data which could not be retrieved, were excluded from the analysis.

### Data Extraction

Two reviewers extracted data using a validated data extraction form following the Users’ Guides to the Medical Literature: A Manual for Evidence-Based Clinical Practice (Guyatt et al., 2015). The extracted data included study method, nationality of the participants, sample size, intervention group, control group, and outcome measures, as well as the first authors, publication year, country of study, participant characteristics, sample size, intervention and control group details, and assessed outcome measures (Table 1).

**Table 1:** Characteristics of Included Studies That Used Cold Compresses (I) or Routine Care (C) in Patients After Transfemoral Percutaneous Coronary Intervention

References/Country	Sample	Intervention Group (IG)	Control Group (CG)	Indicators	Outcome
Baqal & Mahmood (2022)/Iraq	*N*=60 IG (*n*=30) CG (*n*=30) • Age: most 60–69 y • Post-PCI • Size of sheath: 6–7 Fr.	1. Cold compresses: after arterial sheath removal. 2. Ice bag with direct pressure for 20 min and early ambulation after 2 hr complete bed rest.	Standard clinic procedure	Bleeding, hematoma, ecchymosis	Cold compress reduced vascular complications compared with the control group.
Bayındır et al. (2017)/Turkey	*N*=104 IG (*n*=52) • 62.10±13.40 y CG (*n*=52) • 61.60±12.7 y • Post-PCI • Size of sheath: 6–8 Fr.	1. Cold compresses: before arterial sheath removal. 2. Ice bag to the femoral artery puncture site for 20 min before sheath removal.	Standard clinic procedure	Pain (NRS)	Cold compress (3.8±1.4) had lower pain than the control group (5.5±2.1; *p*<.001).
Ebrahimi-Shalmani et al. (2020)/Iran	*N*=110 IG (*n*=55) • 62.36±9.59 y CG (*n*=55) • 61.16±10.78 y • Post-PCI • Size of sheath: no mention	1. Cold compresses: after arterial sheath removal. 2. Icebag (15 °C–18 °C) was placed in position 4 times, each for 20 min at 10-min intervals for 2 hr.	3–4 kg sandbags compression of the femoral artery puncture site for 4 hr	1. Pain (low back pain; VAS) 2. Bleeding, hematoma, ecchymosis	1. Cold compress (4.75±1.71) had lower pain than the control group (7.84±1.01) (*p*<.001). 2. Cold compress had lower hematoma and ecchymosis than the control group at all evaluation times (*p*<.001).
Ginanjar et al. (2018)/Indonesia	*N*=30 IG (*n*=15) CG (*n*=15) • Age: not more than 65 y • Post-PCI • Size of sheath: 6 Fr.	1. Cold compresses: after arterial sheath removal. 2. Ice bag to the puncture site for 20 min and ambulate 10 m 1 hr after sheath removal.	2.5 kg sandbags and immobilization for 6 hr	Bleeding, hematoma	1. There were no significant differences in bleeding between the two groups (*p*=1.000). 2. Cold compresses had lower hematoma than the control group (*p*<.001).
Kareem (2023)/Iraq	*N*=90 IG (*n*=45) CG (*n*=45) • Age: most 50–69 y • Post-PCI • Size of sheath: 6–7 Fr.	1. Cold compresses: after arterial sheath removal. 2. Ice bag for 15–20 min until hemostasis.	Usual nursing care	Bleeding, hematoma, ecchymosis	1. Cold compresses had lower hematoma and bleeding than the control group (*p*<.05). 2. The two groups had no significant differences in ecchymosis (*p*=.078).
Korkmaz & Karagözoğlu (2022)/Turkey	*N*=120 close pad (*n*=40) • 62.75±8.62 y sandbags (*n*=40) • 59.28±9.86 y cold + sandbags (*n*=40) • 60.30±10.54 y • Post-PCI • Size of sheath: no mention	1. Cold compresses: after arterial sheath removal. 2. Ice bag (15 °C–18 °C) for 15 min and sandbag compress 4 hr.	Sandbag compress 4 hr	1. Pain (VAS) 2. Bleeding, hematoma, ecchymosis	1. Cold plus sandbag had lower pain than closed pad and sandbag (*p*=.040). 2. There were no significant differences in bleeding, hematoma, or ecchymosis.
Pamuk & Özkaraman (2024)/Turkey	*N* =210 IG (*n*=105) • 57.28±11.46 y CG (*n*=105) • 58.03±11.39 y • Post-PCI • Size of sheath: 6 Fr.	1. Cold compresses: after arterial sheath removal. 2. 5 kg ice bag (18 °C–20 °C) for 20 min, then at an average temperature of 24.1 °C for 3 hr and 40 min.	5 kg sandbag, average temperature of 24.1 °C for 4 hr	1. Pain (VAS) 2. Bleeding, hematoma, ecchymosis	1. Cold compress (1.50±1.18) had lower pain than the control group (3.45±1.73) (*p*<.001). 2. Cold compress had lower bleeding and ecchymosis than the control group at the second hour (*p*<.005). 3. The two groups had no significant differences in hematoma (*p*=.561).
Sokhanvar et al. (2023)/Iran	*N*=60 IG (*n*=30) • 58.50±9.80 y CG (*n*=30) • 56.70±12.40 y • Post-PCI • Size of sheath: no mention	1. Cold compresses: before arterial sheath removal. 2. 250 g ice bag for 20 min before femoral sheath removal, and 2 kg sandbag for 4–6 hr after sheath removal.	2 kg sandbag for 4–6 hr after sheath removal	Pain (NRS)	Cold compress (0±0) had lower pain than the control group (1.72±0.83; *p*=.005).
Valikhani et al. (2020)/Iran	*N*=60 IG (*n*=30) • 54.90±8.70 y CG (*n*=30) • 56.80±9.80 y • Post-PCI • Size of sheath: no mention	1. Cold compresses: after arterial sheath removal. 2. Ice bag (<100 g) with a sandbag (3 kg) was placed on the site for 15 min, followed by sandbag pressure alone for 45 min. This cycle was repeated four times, totaling 4 hr.	Removed the sheath about 4 hr after the procedure, 3 kg sandbag for 4 hr	Bleeding, hematoma	1. Cold plus sandbag had lower bleeding than the control group (*p*<.050). 2. The two groups had no significant differences in hematoma (*p*=.561).
Valikhani et al. (2023)/Iran	*N*=60 IG (*n*=30) • 54.90±8.70 y CG (*n*=30) • 56.80±9.80 y • Post-PCI • Size of sheath: no mention	1. Cold compresses: after arterial sheath removal. 2. Ice bag (<100 g) with a sandbag (3 kg) was placed at the site for 15 min, followed by sandbag pressure alone for 45 min. This cycle was repeated four times, totaling 4 hr.	Removed the sheath about 4 hr after the procedure, 3 kg sandbag for 4 hr	Pain (NRS)	Cold plus sandbag had lower pain than the control group (*p*<.001).

*Note.* y = year; PCI = percutaneous coronary intervention; Fr. = French; NRS = Numerical Rating Scale; VAS = Visual Analogue Scale.

### Assessment of Study Quality

Two reviewers independently assessed the methodological quality of the included RCTs using the Joanna Briggs Institute (JBI) Critical Appraisal Checklist for Randomized Controlled Trials (Tufanaru et al., 2022). This tool consists of 13 items in the five domains of randomization, allocation concealment, blinding, completeness of follow-up, and appropriateness of statistical analysis. Each item is rated Yes, No, Unclear, or Not applicable. For descriptive purposes, one point is assigned for each “Yes” response, yielding a possible total score range of 0–13. Studies scoring <7 were considered low quality and excluded from the analysis. Any disagreement between the reviewers was resolved through consultation with a third reviewer.

### Data Analysis

Meta-analyses were conducted separately for continuous and binary outcomes. For continuous outcomes, standardized mean differences (SMDs) with 95% confidence intervals (CIs) were calculated using the Hedges’ g method to adjust for small-sample bias (Hedges & Olkin, 1985). The meta-analyses were performed using a random-effects model, with the DerSimonian–Laird estimator applied to assess between-study heterogeneity (τ²; DerSimonian & Laird, 1986). To improve the accuracy of the confidence intervals, the Hartung–Knapp adjustment was applied (Hartung & Knapp, 2001).

For binary outcomes, the risk ratios (RRs) and corresponding 95% CIs were calculated using the Mantel–Haenszel method (Greenland & Robins, 1985). Continuity correction of 0.5 was applied only to included studies with zero events in one arm. As with the continuous outcomes, random-effects models and the DerSimonian–Laird method were used for τ² estimation.

Between-study heterogeneity was assessed using the Cochran’s Q test and *I*² statistic. To further investigate potential sources of heterogeneity, subgroup analyses were conducted based on whether a cold compress was administered alone or in combination with other treatments. Also, potential publication bias was assessed separately for continuous and binary outcomes, funnel plots were visually inspected for asymmetry, and small-study effects were formally tested using Egger’s test (Egger et al., 1997).

All analyses were conducted using the meta package in R (version 4.4.1), and effect estimates were stratified by outcome type (Balduzzi et al., 2019).

## Results

**Figure 1:**
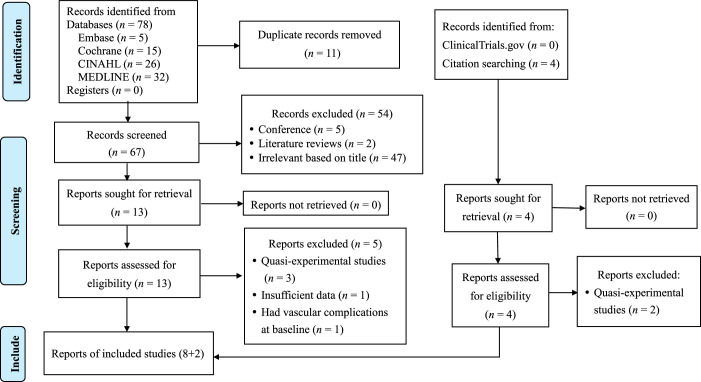
Preferred Reporting Items for Systematic Reviews and Meta-Analyses

Of the 82 articles initially identified, 78 were retrieved from the database searches, and four were retrieved from other sources. After 11 duplicate records were removed using EndNote, 67 articles were excluded based on title and abstract screening, leaving 17 articles (13 from databases and four from other sources) for full-text review. Ultimately, 10 articles met the inclusion criteria and were included in this meta-analysis (eight from the searched databases and two from other manual searches; Figure 1).

### Study Characteristics

The ten studies included in the analysis were all RCTs conducted in Asia between 2017 and 2024. The total sample size was 904, with individual studies enrolling between 30 and 210 participants. The analysis incorporated the following elements (see Table 1).

#### Sheath Size

Five of the studies did not specify arterial sheath size (Ebrahimi-Shalmani et al., 2020; Korkmaz et al., 2022; Sokhanvar et al., 2023; Valikhani et al., 2020; Valikhani et al., 2023). The sizes used in the five studies that specified size were 6 French (Ginanjar et al., 2018; Pamuk and Özkaraman, 2024), 6–7 French (Baqal et al., 2022; Kareem et al., 2023), and 6–8 French (Bayındır et al., 2017).

#### Timing of Cold Compress Application

In two of the included studies, cold compresses were applied before arterial sheath removal (Bayındır et al., 2017; Sokhanvar et al., 2023), while in the other studies, the intervention was administered after sheath removal (Baqal et al., 2022; Ebrahimi-Shalmani et al., 2020; Ginanjar et al., 2018; Kareem et al., 2023; Korkmaz et al., 2022; Pamuk & Özkaraman, 2024; Valikhani et al., 2020; Valikhani et al., 2023).

#### Temperature of Cold Compress Application

Only three of the included studies specified that the temperature of the cold compress be maintained between 15 °C and 20 °C (Ebrahimi-Shalmani et al., 2020; Korkmaz & Karagözoğlu, 2022; Pamuk et al., 2024).

#### Weight and Duration of Cold Compress Application

The weight and duration of the cold compress application varied across the included studies. Most of the interventions involved applying the cold compress for 15–20 min per session, with one study repeating this application over a period of up to 2 hr (Ebrahimi-Shalmani et al., 2020). Most of the included studies did not report the weight of the ice bags used, with only Pamuk et al. (2024), Sokhanvar et al. (2023), and Valikhani et al. (2020, 2023), respectively, specifying that a 5 kg ice bag, a 250 g ice bag, and an ice bag weighing <100 g be used.

### Methodological Quality Assessment

The methodological quality of the included RCTs, assessed using the JBI Critical Appraisal Checklist (13 items, maximum score 13), ranged from 9 to 12, indicating overall moderate to high quality. While most of the included studies reported adequate randomization and statistical analyses, none implemented blinding for participants, providers, and outcome assessors simultaneously (Table 2).

**Table 2:** JBI Critical Appraisal Checklist for Randomized Controlled Trials

Questions Studies	Q1	Q2	Q3	Q4	Q5	Q6	Q7	Q8	Q9	Q10	Q11	Q12	Q13	Score
Baqal & Mahmood (2022)	Yes	Yes	Yes	No	Unclear	Unclear	Yes	Yes	Yes	Yes	Yes	Yes	Yes	10
Bayındır et al. (2017)	Yes	Yes	Yes	No	Unclear	Unclear	Yes	Yes	Yes	Yes	Yes	Yes	Yes	10
Ebrahimi-Shalmani et al. (2020)	Yes	Yes	No	No	Unclear	Unclear	Yes	Yes	Yes	Yes	Yes	Yes	Yes	9
Ginanjar et al. (2018)	Yes	Yes	Unclear	No	Unclear	Unclear	Yes	Yes	Yes	Yes	Yes	Yes	Yes	9
Kareem et al. (2023)	Yes	Yes	Yes	No	Unclear	Unclear	Yes	Yes	Yes	Yes	Yes	Yes	Yes	10
Korkmaz & Karagözoğlu (2022)	Yes	Yes	Yes	No	Unclear	Unclear	Yes	Yes	Yes	Yes	Yes	Yes	Yes	10
Pamuk & Özkaraman (2024)	Yes	Yes	Yes	No	No	Yes	Yes	Yes	Yes	Yes	Yes	Yes	Yes	11
Sokhanvar et al. (2023)	Yes	Yes	Yes	No	Yes	Yes	Yes	Yes	Yes	Yes	Yes	Yes	Yes	12
Valikhani et al. (2020)	Yes	Yes	Yes	No	Unclear	Unclear	Yes	Yes	Yes	Yes	Yes	Yes	Yes	10
Valikhani et al. (2023)	Yes	Yes	Yes	No	Unclear	Unclear	Yes	Yes	Yes	Yes	Yes	Yes	Yes	10

*Note.* JBI = The Joanna Briggs Institute. Q1: Was true randomization used to assign participants to treatment groups? Q2: Was allocation to treatment groups concealed? Q3: Were treatment groups similar at the baseline? Q4: Were participants blind to treatment assignment? Q5: Were those delivering treatment blind to treatment assignment? Q6: Were outcome assessors blind to treatment assignment? Q7: Were treatment groups treated identically, other than the intervention of interest? Q8: Was follow-up complete, and if not, were differences between groups in terms of their follow-up adequately described and analyzed? Q9: Were participants analyzed in the groups to which they were randomized? Q10: Were outcomes measured in the same way for treatment groups? Q11: Were outcomes measured in a reliable way? Q12: Was an appropriate statistical analysis used? Q13: Was the trial design appropriate, and were any deviations from the standard RCT design (individual randomization, parallel groups) accounted for in the conduct and analysis of the trial?

### Meta-Analysis Results

#### Pain

Four of the RCTs (*n*=434) evaluated the effect of cold compress use on puncture-site pain, with the pooled analysis indicating a significant reduction in pain compared with routine care (SMD=−1.56, 95% CI [−2.86, −0.25]). However, substantial heterogeneity was observed (Q=20.7, *df*=3, *p*<.01; *I*²=85.5%; τ²=0.32).

**Figure 2:**
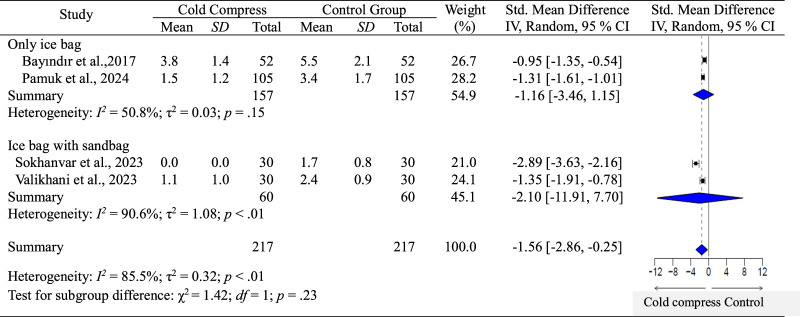
Forest Plot of Pain for Cold Compress or Control

Subgroup analysis stratified by intervention type demonstrated that studies in which ice bags only were applied (2 RCTs, *n*=314) showed a consistent trend toward pain reduction (SMD=−1.16, 95% CI [−3.46, 1.15]), with moderate heterogeneity (*I*²=50.8%). In contrast, the studies in which ice bags and sandbags were applied (2 RCTs, *n*=120), while also showing pain reduction (SMD=−2.10, 95% CI [−11.91, 7.70]), were affected by significant heterogeneity (*I*²=90.6%). No statistically significant difference among subgroups was identified (χ²=1.42, *df*=1, *p*=.23), indicating both intervention modalities reduced pain. However, the magnitude and consistency of the effects could not be conclusively distinguished due to the wide confidence intervals and variability observed across the trials (Figure 2).

#### Vascular Complications

**Figure 3:**
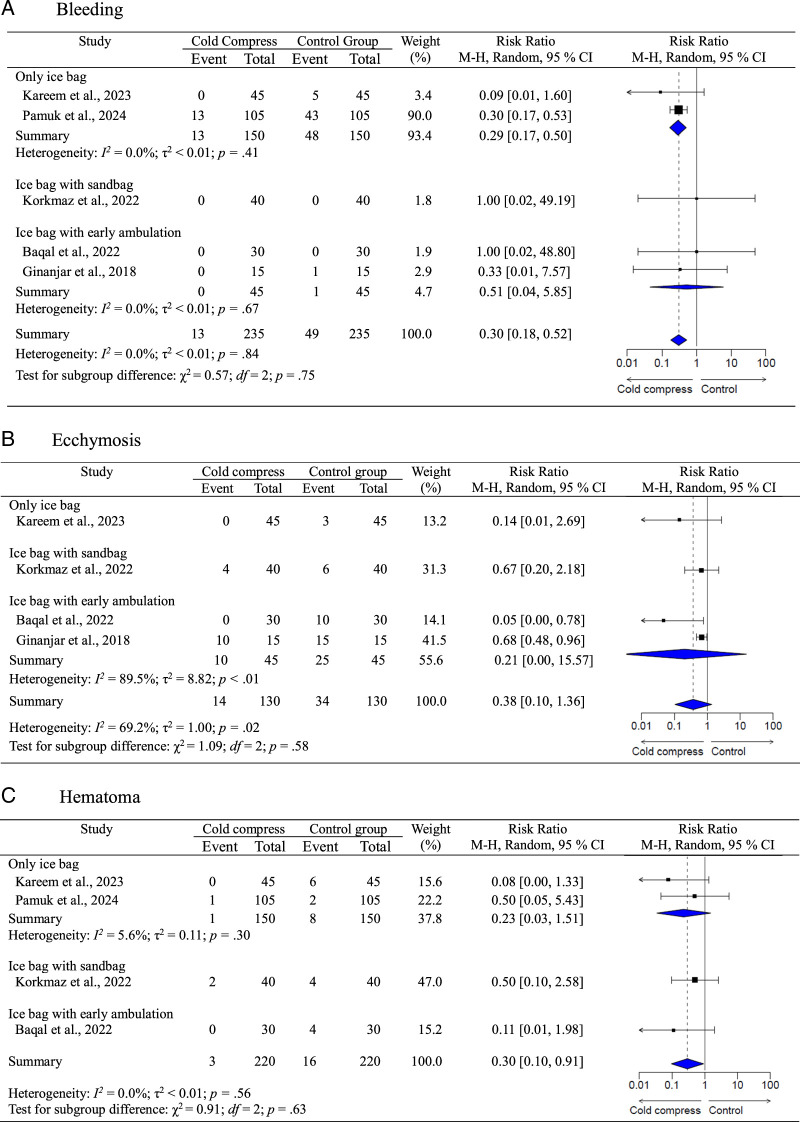
Forest Plot of Vascular Complications for Cold Compress or Control

*Bleeding.* Five of the included studies (*n*=470) considered bleeding outcomes, with the pooled analysis showing that cold compress application reduced the risk of bleeding significantly more than routine care (RR=0.30, 95% CI [0.18, 0.52]). Heterogeneity across the included studies was negligible (Q=1.42, *df*=4, *p*=.84; *I*²=0.0%; τ²<0.01). The subgroup analyses stratified by intervention type (ice bag alone, ice bag combined with sandbag, and ice bag with early ambulation) revealed no significant subgroup differences (*p*=.75; Figure 3).

#### Ecchymosis

Four of the included studies (*n*=260) considered ecchymosis, with the pooled estimate indicating a nonsignificant reduction in this symptom (RR=0.38, 95% CI [0.10, 1.36].

Moderate-to-high heterogeneity was observed across studies (Q=9.74, *df*=3, *p*=.02; *I*²=69.2%; τ²=1.00). Subgroup analyses according to intervention modality (ice bag alone, ice bag combined with sandbag, and ice bag with early ambulation) suggested that studies involving ice bag application with early ambulation contributed substantially to the observed heterogeneity (*I*²=89.5%). However, no statistically significant subgroup differences were identified (*p*=.58; Figure 3).

#### Hematoma

Four of the included studies (*n*=440) assessed hematoma, with cold compress application associated with a significant reduction in hematoma incidence (RR=0.30, 95% CI [0.10, 0.91]). No heterogeneity was detected (Q=2.05, *df*=3, *p*=.56; *I*²=0.0%; τ²<0.01). Subgroup analyses according to intervention type (i.e., only ice bag, ice bag with sandbag, and early ambulation plus ice bag) showed generally consistent results across groups, with no evidence of subgroup differences (*p*=.63; Figure 3).

#### Publication Bias

Given the limited number of studies per outcome (<10), funnel plot symmetry could not be reliably assessed. Therefore, Egger’s regression test was performed instead, the results of which did not indicate significant publication bias for pain (*p*=.34), hematoma (*p*=.17), bleeding (*p*=.73), or ecchymosis (*p*=.16).

## Discussion

This systematic review and meta-analysis support that cold compresses reduce pain and vascular complications significantly compared with routine care. The included studies were conducted in Asian populations, primarily involving patients aged 50 to 70 undergoing transfemoral PCI. Notably, the geographic homogeneity of the included studies may limit the generalizability of the findings to populations in other regions, where cultural norms, patient care practices, and health care system structures may differ (Khan & Ludman, 2022; Su et al., 2024). Cross-cultural variations may influence the implementation and effectiveness of cold compress interventions used in PCI care. Therefore, future multicenter randomized controlled trials with standardized intervention protocols across diverse cultural and health care contexts are warranted to validate the findings of this study and establish optimal application parameters.

The pooled analyses conducted in this study show cold compresses as effective in reducing pain intensity, bleeding, and hematoma, while exhibiting a nonsignificant positive effect on ecchymosis. The subgroup analyses provided further insights, with studies applying ice bags alone demonstrating more consistent analgesic effects (*I*²=50.8%) and studies applying ice bags and sandbags in combination or practicing early ambulation showing higher variability (*I*² > 85%). Although the between-group differences were not statistically significant, these findings suggest adjunctive strategies, such as additional compression or mobilization, may increase outcome variability. For vascular complications, the protective effects on bleeding and hematoma of using a cold compress were consistent across all subgroups, with negligible heterogeneity. In contrast, the results for ecchymosis exhibited moderate to high heterogeneity, particularly in those studies in which a cold compress was combined with early ambulation, which may explain the lack of statistical significance (RR=0.38, 95% CI [0.10, 1.36]). Clinically, these observations highlight the importance of standardizing intervention protocols, including whether a cold compress should be applied independently or in combination with adjunctive measures.

The beneficial effects of cold compresses may be explained by their physiological mechanisms. Vasoconstriction reduces arterial and capillary blood flow, which promotes platelet aggregation, decreases vascular permeability, and slows nerve conduction, thereby reducing pain perception and vascular complications (Asy’ary et al., 2024; Halm et al., 2022). These mechanisms help stabilize the puncture site, minimize post-sheath removal complications, and improve patient comfort and recovery satisfaction. However, methodological inconsistencies across trials in terms of, for example, compress weight (ranging from <100 g to 5 kg), application timing (before vs. after sheath removal), and lack of standardized temperature reporting may have influenced study outcomes and should be addressed in future research.

The findings of this study expand upon and complement the descriptive synthesis of Sugiharto et al. (2025). Their review focused primarily on hematoma and pain, while this study additionally evaluated ecchymosis and bleeding, offering a broader understanding of clinical outcomes. Moreover, the pooled effect estimates and heterogeneity measures generated by the quantitative meta-analysis in this study are not provided in their work. Also, the subgroup analyses conducted in this study uniquely clarified potential sources of variability, effectively addressing a key limitation of prior reviews. Finally, in following a preregistered protocol, applying PRISMA standards, and using the JBI checklist, this study effectively improved transparency, reproducibility, and clinical applicability. Collectively, these contributions strengthen the evidence base for cold compress use in post-PCI care and provide more robust guidance for nursing practice.

### Limitations

This study was affected by several limitations. First, all of the included randomized controlled trials were conducted in Asian countries and primarily involved patients aged 50–70 undergoing transfemoral PCI. Thus, the high level of geographic and demographic homogeneity within the sample may limit the generalizability of the findings to populations in other cultural and health care contexts. Although the subgroup analyses targeting intervention modality (i.e., ice bag alone, ice bag plus sandbag, and ice bag with early ambulation) were performed to partly address this limitation, further multicenter studies across diverse regions are required to confirm the generalizability of the findings. Second, notable variations in cold compress protocols were identified across the 10 included studies in terms of temperature (therapeutic vs. unspecified), duration and frequency of application, timing relative to sheath removal, and compress weight. These variations may have contributed to clinical heterogeneity. Although the subgroup analyses helped clarify some of these inconsistencies, more standardized intervention protocols are needed to further minimize variability in future trials.

Third, comprehensive reporting of procedural details such as sheath size, activated clotting time values at removal, and whether manual compression was applied before cold compress use was absent from several of the included studies. This issue limited comparability across studies, potentially influencing the observed outcomes.

Fourth, none of the included studies implemented blinding of participants, health care providers, or outcome assessors. This issue has been reported to increase the risk of performance and detection bias, particularly for subjective outcomes such as pain intensity.

Fifth, the small number of studies per outcome restricted the statistical power for certain analyses, although subgroup analyses were conducted whenever feasible. Moreover, publication bias was formally assessed using Egger’s test, which did not identify significant asymmetry. However, the limited number of trials reduced the robustness of the results. Future meta-analyses using larger datasets are needed to confirm the stability of the findings and allow for more comprehensive sensitivity analyses.

Finally, most of the included studies focused on short-term outcomes such as immediate postprocedural pain and vascular complications. The long-term effects of cold compress application after transfemoral PCI remain unclear and should be investigated in future longitudinal studies.

### Conclusions

The evidence provided by this meta-analysis supports using cold compresses as an effective nonpharmacological intervention to alleviate pain and reduce vascular complications such as bleeding, ecchymosis, and hematoma following transfemoral PCI. Despite variations in intervention parameters, the overall findings suggest cold compress therapy is a safe, simple, and cost-effective adjunct to standard post-PCI care. Application method standardization in terms of timing, temperature, weight, and duration should be a focus of future research to both optimize clinical efficacy and ensure consistent implementation across care settings.

### Implications

The findings of this meta-analysis indicate cold compresses to be an effective, low-cost, and nonpharmacological intervention that may help alleviate pain and reduce vascular complications in patients following transfemoral PCI. The results support the potential integration of cold compresses into routine postprocedural care to enhance patient comfort and improve recovery outcomes. However, given the variability in the application protocols used across the included studies, it is critical that clinical settings develop standardized guidelines tailored to their specific patient populations and available resources. Nurses and health care providers may consider incorporating cold compresses into their care plans and taking measures to ensure proper patient assessment and monitoring during application. Future multicenter studies must refine these protocols and establish evidence-based recommendations for optimal cold compress use in post-PCI care.

### Author Contributions

Study conception and design: Both authors

Data collection: Both authors

Data analysis and interpretation: YCL

Drafting of the article: Both authors

Critical revision of the article: Both authors

## Supplementary Material
