# Supplementary material for: Effects of Cold Compress on Pain and Vascular Complications in Patients With Percutaneous Coronary Intervention: A Systematic Review and Meta-Analysis
Source: J Nurs Res. 2026 Aug 13;34(4):e474. doi: 10.1097/jnr.0000000000000756 (PMC13465689; doi:10.1097/jnr.0000000000000756)
Supplement: Supplementary file 1 [file jnr-34-e474-s001.docx]

**Supplementary file 1**

Search Strategy

| **Database** | **#** | **Search Syntax** |
| --- | --- | --- |
| 1. Embase | 1 | (transfemoral percutaneous coronary interventions OR coronary interventions OR cardiac catheterization OR post cardiac catheterization):ti,ab,kw |
|  | 2 | "percutaneous coronary interventions"/exp |
|  | 3 | ("cold pack" OR "cold compress" OR "ice-bag" OR "cold sand pack" "cold therapy"):ti,ab,kw |
|  | 4 | "cold compress"/exp |
|  | 5 | (vascular complications OR adverse reactions OR pain OR hematoma OR bleeding OR ecchymosis OR hemastasis):ti,ab,kw |
|  | 6 | " complications "/exp OR "hemostasis"/exp |
|  | 7 | (#1 OR #2) AND (#3 OR #4) AND (#5 OR #6) AND [embase]/lim |
| 2.MEDLINE (Ovid) | 1 | (transfemoral percutaneous coronary interventions OR coronary interventions OR cardiac catheterization OR post cardiac catheterization).mp |
|  | 2 | Exp " percutaneous coronary interventions "/ |
|  | 3 | ("cold pack" OR "cold compress" OR "ice-bag" OR "cold sand pack" OR "cold therapy").mp |
|  | 4 | exp "cold therapy"/ |
|  | 5 | (vascular complications OR adverse reactions OR pain OR hematoma OR bleeding OR ecchymosis).mp |
|  | 6 | exp "complications"/ OR exp "hemostasis" |
|  | 7 | (1 OR 2) AND (3 OR 4) AND (5 OR 6) |
| 3. Cochrane | 1 | (transfemoral percutaneous coronary interventions OR coronary interventions OR cardiac catheterization OR post cardiac catheterization):ti,ab,kw |
|  | 2 | [mh "percutaneous coronary interventions"] |
|  | 3 | ("cold pack" OR "cold compress" OR "ice-bag" OR "cold sand pack" OR "cold therapy"):ti,ab,kw |
|  | 4 | [mh "cold therapy"] |
|  | 5 | (vascular complications OR adverse reactions OR pain OR hematoma OR bleeding OR ecchymosis):ti,ab,kw |
|  | 6 | [mh "hemostasis"] |
|  | 7 | (#1 OR #2) AND (#3 OR #4) AND (#5 OR #6) |
| 4. CINAHL | 1 | transfemoral percutaneous coronary interventions OR coronary interventions OR cardiac catheterization OR post cardiac catheterization |
|  | 2 | mh ("percutaneous coronary interventions ") |
|  | 3 | "cold pack" OR "cold compress" OR "ice-bag" OR "cold sand pack" OR "cold therapy" |
|  | 4 | mh ("cold compress+") |
|  | 5 | vascular complications OR adverse reactions OR pain OR hematoma OR bleeding OR ecchymosis |
|  | 6 | mh ("complications +") |
|  | 7 | (S1 OR S2) AND (S3 OR S4) AND (S5 OR S6) |
